# Voltammetric determination of acetaminophen in pharmaceutical preparations and human urine using glassy carbon paste electrode modified with reduced graphene oxide

**DOI:** 10.1007/s44211-022-00150-2

**Published:** 2022-07-09

**Authors:** Amir Shaaban Farag

**Affiliations:** Department of Analytical Chemistry, Faculty of Pharmacy, Helwan University, Helwan, 11795 Cairo Egypt

**Keywords:** Glassy carbon paste electrode, Drug analysis, Reduced graphene oxide, Square wave voltammetry, Acetaminophen, urine analysis

## Abstract

A completely new direct voltammetric method has been developed for determination of acetaminophen (APAP), known as popular analgesic drug. The present electroanalytical method is based on anodic oxidation of APAP at the glassy carbon paste electrode modified with reduced graphene oxide (RGO). Key experimental conditions were studied, resulting in a set of optimal conditions: acetate buffer (pH 5.0) as working medium electrolyte, content of RGO, parameters of squarewave voltammetry including the potential step of 5 mV, potential amplitude of 50 mV, and frequency of 40 Hz. If peak area is used for evaluation, a linear range from 1.2 × 10^–6^ to 2.2 × 10^–4^ mol L^−1^ characterized by determination coefficient of 0.9971, limits of quantification and detection, 9.3 × 10^–7^ mol L^−1^ and 3.1 × 10^–7^ mol L^−1^, respectively, will be obtained. Under validation, the precision was described by relative standard deviation of 2.9% for the model sample analysis. Finally, the developed voltammetric method was compared with a reference high-performance liquid chromatography method in the analysis of commercially available pharmaceutical preparation and human urine collected from five healthy volunteers.

**Graphical abstract:**

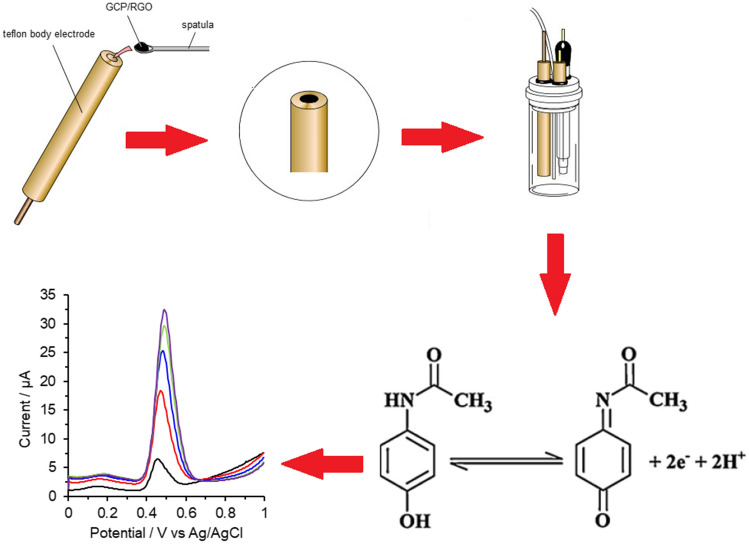

**Supplementary Information:**

The online version contains supplementary material available at 10.1007/s44211-022-00150-2.

## Introduction

Acetaminophen (APAP), also known as paracetamol, represents the most common analgesic used worldwide and recommended as the first-line therapy in pain conditions by the World Health Organization (WHO) [1]. This medicament can be also used for its antipyretic effect, helping to reduce fever [2], being available in a variety of forms including a syrup form, regular tablet, effervescent tablet, injection, suppository, and other forms [3].

APAP is the major metabolite of phenacetin and acetanilide [2] and mainly metabolized in the liver by 1st order kinetics by three different pathways: conjugation with glucuronide, conjugation with sulfate, and oxidation through the cytochrome P450 enzyme pathway, mainly CYP2E1 to produce a reactive metabolite (*N*-acetyl-*p*-benzoquinone imine; NAPQI). High doses of APAP (overdoses) can lead to a hepatic necrosis due to the depletion of glutathione and binding of high levels of the reactive metabolite NAPQI to important parts of liver cells. The afore-mentioned damage of liver can be prevented by the early administration of sulfhydryl compounds, e.g., methionine or *N*-acetylcysteine [4]. Less than 5% of the total amount is excreted in urine as free (unconjugated) APAP and 90% of the administered dose is excreted within 24 h [2]. In other literature [5], it is possible to learn that about 2% APAP is excreted unchanged; therefore, for 250 mg dose of APAP recommended for children, a minimum detected concentration of ~ 30 μmol per liter of urine might be expected.

Regarding all the facts mentioned, determination of APAP at various concentration levels is of great clinical importance. A wide variety of analytical methods have been developed for identification and quantification of APAP in real samples (biological fluids and pharmaceutical preparations), employing colorimetry [6], various chromatographic [7–10], or electroanalytical (voltammetric) methods [11–16]. In addition, several amperometric biosensors, utilizing aryl-acylamidase, horseradish peroxidase or tyrosinase have been developed [5, 17–19].

Graphene, a special 2D-form of carbon (with atoms in the sp2 bonds), has captured great attention in the development of modified electrochemical electrodes because of its large surface area, high electrical conductivity, almost perfect capacitance and ingenuity [20, 21]. It can give rise to graphene oxide (GO) and its reduced form (RGO); the latter being particularly suitable for electrochemical sensors due to a higher conductivity than the original GO and preparable by chemical [22] or even electro-chemical [23] reduction.

This contribution presents a new voltammetric method for the determination of APAP in tablet dosage forms and human urine which is based on anodic oxidation of APAP to NAPQI with the participation of two protons and two electrons [24] at glassy carbon paste electrode (GCPE) modified with 10% (*w*/*w*) RGO in acetate buffer of pH 5. Its principles, optimization of key experimental conditions, as well as all important electroanalytical characteristics with a validation by reference method based on reversed-phase high-performance liquid chromatography with spectrophotometric detection (RP–HPLC/UV–Vis) [25, 26], are described in the following sections.

## Experimental

### Reagents and chemicals

Analytical standards of ≥ 99% acetaminophen, ≥ 99.8% acetic acid, ≥ 85% phosphoric acid, ≥ 99.5% boric acid, and a 10 gm commercial package of reduced graphene oxide (RGO) were purchased from Sigma–Aldrich (Cairo, Egypt). Other reagents, namely: ascorbic acid, ≥ 99% uric acid, sodium acetate, and sodium hydroxide (all *p.a.* grade) were purchased from Lach-Ner (Neratovice, Czech Republic). Ultrapure water was used throughout the work when obtained by passing of the already deionized water through a purification unit ("Milli-Q" system; Merck-Millipore, Darmstadt, Germany).

### Preparation and pretreatment of the glassy-carbon paste electrode

The glassy-carbon paste electrode, serving as a substrate for the proper electrode (with the layer of RGO), was prepared by thoroughly hand-mixing of 70%(v/v) glassy carbon micro particles, 20%(v/v) paraffin oil, and 10%(v/v) reduced graphene oxide.

The resultant paste-like mixture was packed into a piston-driven carbon paste holder with Teflon^®^ body (and a cavity of 20 mm in diameter). Whenever needed, the paste in the filled body could be extruded and the active surface renewed by smoothing it with a wet filter paper until a shiny appearance. Then, the working electrode of RGO/GCPE type was ready for electrochemical measurement(s).

### Instrumentation and accessories

All electrochemical measurements were carried out in a 50 mL glass cell fixed in mounted head with three-electrode system. It consisted of the working electrode (RGO/GCPE if not stated otherwise), Ag/AgCl/3 M KCl reference and Pt-sheet auxiliary electrodes; all being in connection with an electrochemical analyser (model PGSTAT101, Autolab-Metrohm; Prague, Czech Rep.) that had been controlled by the software ("Nova 1.11" version, Autolab). Whenever needed, the pH values were measured with a pH meter, the glass electrode, and a series of the standards for calibration in an interval of pH 1–11 (all from Metrohm; Prague, Czech Republic).

### Square-wave voltammetry

Square-wave voltammetry (SWV) of the analyte of interest provided a single well-developed oxidation peak at about + 0.5 V vs. ref. in an acetate buffer (AcB, pH 5), when applying a potential scan from 0.0 to + 1.0 V at a potential step of 2.5 mV, potential amplitude of 25 mV, and a frequency 40 Hz. The quantification of APAP in tablet dosage forms and in real samples was performed using the calibration-curve method (in an interval of 4–220 μmol L^−1^ APAP). Each analysis was repeated minimally five times and the respective results are shown as confidence intervals.

### Analysis of the tablet dosage form

The calibration curve from 20 to 100 μmol L^−1^ was used to determine APAP amount in the tablets of a commercial product ("Panadol^®^500 mg", purchased in an Egyptian pharmacy); each being grinded in a ceramic mortar, mixed, and accurately weighed as the amount equivalent to 1.0 × 10^−2^ M APAP after dissolution in a 250 mL distilled water. Afterwards, the solution prepared was sonicated for 30 min, filtered, and different dilutions within the corresponding linear range prepared.

An excellent recovery of 99.3% was achieved, indicating the proper accuracy and reliability of the new method and its suitability to quantify APAP in tablet dosage forms and related samples in a simple and rapid way.

### Analysis of the urine real sample

**Fig. 1:**
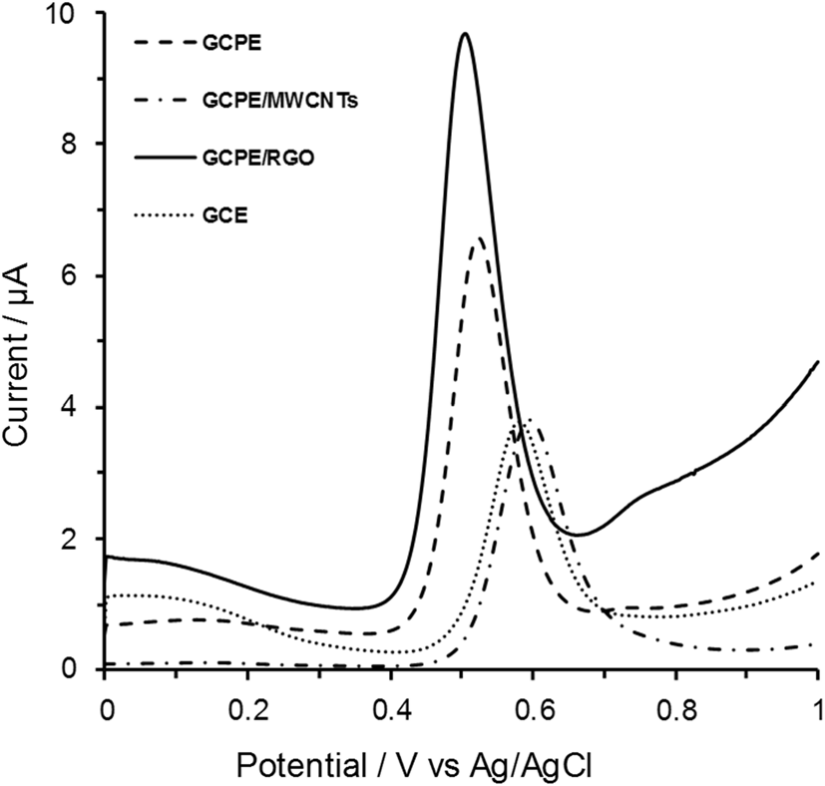
Square wave voltammograms of 100 μmol L^−1^ APAP at different bare and surface modified electrodes in 0.1 mol L^−1^ acetate buffer solution pH 5.0

As already mentioned, the real sample was taken from five volunteers. More specifically, the sample was collected after 4-h ingestion when the volunteers had taken a pill containing a dosage of 500 mg APAP. The procedure described in detail in the Experimental section was applied in five replicates. Typical voltammograms of the respective analysis are illustrated in Fig. 1S.

### Reference chromatographic method

Chromatographic analysis in the reverse-phase HPLC mode and with gradient elution was performed with a system comprising LC-20ADXR binary gradient pump, DGU-20 degassing unit, an SPD-M30A DAD, an SIL-20 AC XR autosampler (all from Shimadzu, Kyoto, Japan), and LCO 102 column thermostat (Ecom, Prague, Czech Rep.). A C18 column ("Ascentis Express" type; 150 mm × 3.0 mm, 2.7 μm) was chosen for achieving the optimum separation at a detection wavelength of 243 nm. The mobile phase composition was 0.3% formic acid in water (A) and methanol (B) with a gradient-elution program from 20 to 40% B for 10 min and at constant temperature of 30 °C. The flow rate was 0.5 mL min^−1^ and injection volume of 5 μL.

### Statistical evaluation

All analyses of selected samples were always made in five replicates (*n* = 5) and the final results calculated and presented as the confidence intervals *x̄* ± *st*_1−*α*_ (where *x̄* is the arithmetic mean, *s* the standard deviation, and *t*_1−*α*_ the critical value 2.015 of Student's *t* distribution for five repetitions at a significance level *α* = 0.05 (with 95% probability).

## Results and discussion

### Electrochemical behaviour of acetaminophen

The aim of work is to present a time saving with simple steps procedure with considerable accuracy and sensitivity that can be applied successfully on real biological samples to have an advantage over already published methods dealing with electrochemical determination of APAP using RGO as they include multi-step and complicated working electrode preparation procedures requiring up to 4 h and limited to spiked samples only [27–31]. Initial SWV measurements were performed in a test solution of 100 μmol L^−1^ APAP in AcB (pH 5.0) with a series of electrodes, namely, the bare glassy carbon electrode (GCE, a commercial product by Metrohm), bare glassy-carbon paste electrode and its two modified variants: either with multi-walled carbon nanotubes (GCPE and MWCNT/GCPE, resp.), or with the reduced graphene oxide, i.e., RGO/GCPE.

The electrochemical oxidation of APAP gave rise to a single well-defined peak at about + 0.5 V vs. ref. with the bare GCPE and RGO/GCPE and approximately at + 0.6 V when using the GCE or MWCNT/GCE. By comparison of the actual performance at the four electrodes tested it was found that, the most satisfactory signal was obtained for the GCPE/RGO as documented by the corresponding voltammogram in Fig. 1. Therefore, the last-named configuration was the (working) electrode of choice and used in all further measurements.

### Effect of pH

Surveying the literature revealed that, APAP is an electrochemical oxidized in a pH-dependent two-electron, two-proton process to *N*-acetyl-*p*-quinon-imine (NAPQI).

**Fig. 2:**
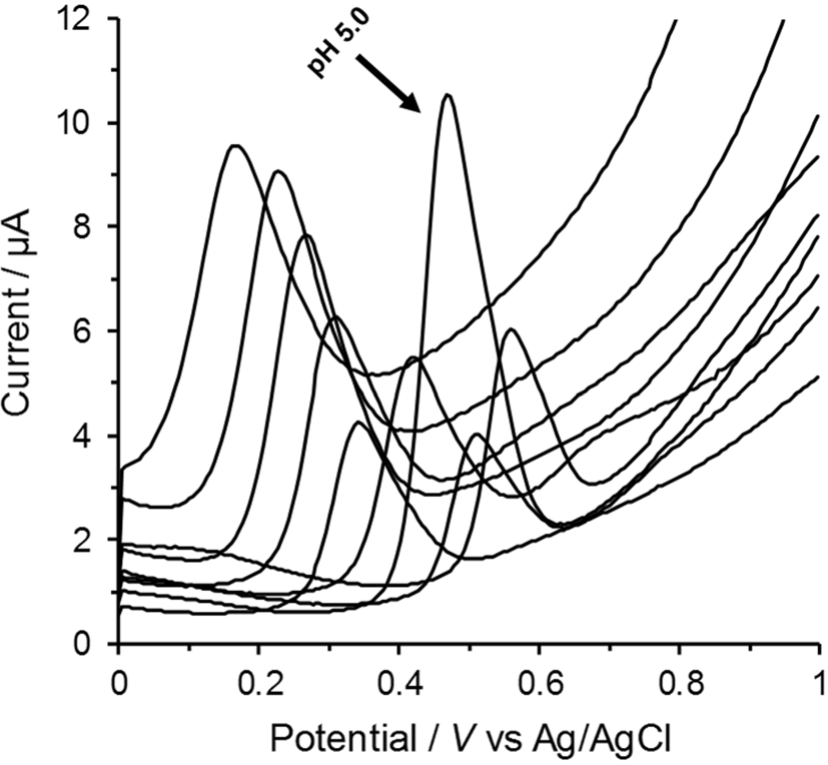
Square wave voltammograms of 100 μmol L^−1^ APAP measured using Britton–Robinson buffer in the pH range (3–11) at GCPE/RGO

The electrochemical oxidation of acetaminophen in various pHs using cyclic voltammetry showed that electrochemically generated NAPQI participates in different type reactions based on solution’s pH. It is hydrolyzed in strong acidic media (pH < 5) and hydroxylated in strong alkaline media (pH > 9) [32]. It has been also reported that the stability of NAPQI is influenced by the sample pH with the highest stability in the pH range 4–9 [33, 34]. The pH of a solution affects the electrochemical reaction by shifting the redox potential to more positive or negative directions with changing peak currents response due to the variation of acid dissociation of APAP [35]. The pH-study was performed with a set of Britton–Robinson buffers covering the range of pH 3–11. When considering the overall size and shape of the oxidation peak, as well as the background currents level within the potential range applied (see Fig. 2), the best response was achieved at pH 5.0 used as optimum pH in all next experiments.

### Optimization of the parameters of square-wave voltammetric ramp

Selection of optimal SWV conditions was made based on the respective series of comparative measurements, when the effects of potential step, amplitude and frequency were investigated individually at the constant settings of all the remaining parameters.

#### Performance of the working electrode

During the characterization of the RGO/GCPE working electrode, it has been ascertained that there is a very favourable level of background currents, as well as no distinct memory effects after repeated scans (for both, see Fig. 2S).

**Fig. 3:**
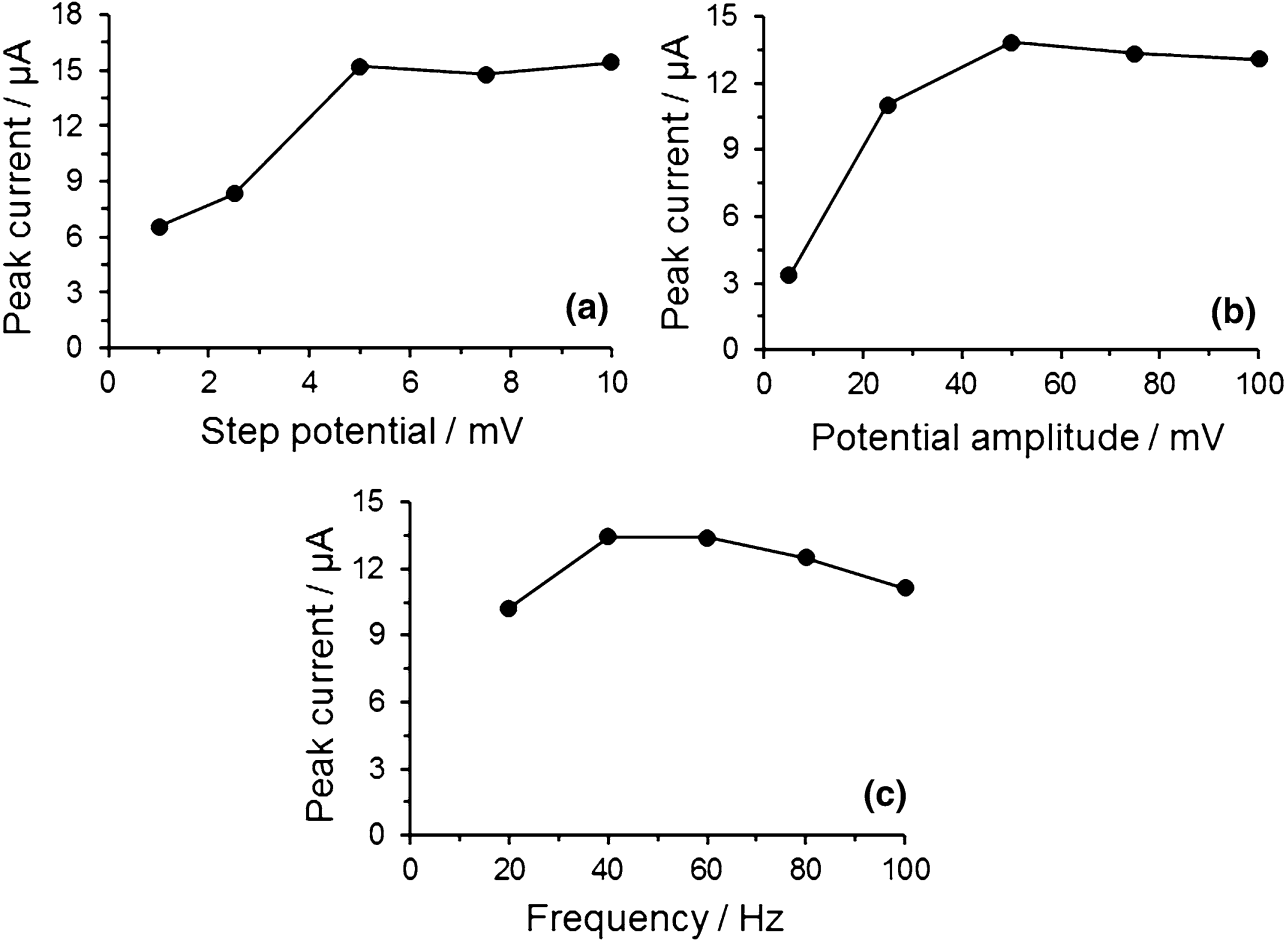
Dependence of peak current response of 100 μmol L^−1^ APAP on **a** step potential, **b** potential amplitude and **c** frequency measured in 0.1 mol L^−1^ acetate buffer solution pH 5.0 at GCPE/RGO

*Note:* The effect of the content of methanol on the electrode response was studied as well, because the use of this organic solvent could improve the solubility of the analyte at high concentrations. However, as known, carbon paste-like electrodes may undergo disintegration in such methanol-containing media [36] although glassy carbon-based mixtures are generally more resistant. Thus, the resistivity of the actual paste was examined in a series of electrolytes having contained 10, 20, 30, 40, and 50%(v/v) methanol in 0.1 mol.L^−1^ AcB. As expected, it was found that the most satisfactory response could be achieved in the methanol-free buffer (Fig. 3S).

#### Potential step

The potential step effect was studied while setting potential amplitude and frequency at usually chosen values; i.e., 25 mV and 40 Hz, respectively. Figure 3a reveals that the potential-step value of 5 mV has been the optimum for the determination of APAP; here, the criterion of choice being the highest current obtained.

#### Potential amplitude

Figure 3b shows the effect of potential amplitude investigated over the range of 5–100 mV. With the increasing values of potential amplitude, the peak heights recorded had exhibited a significant increase up to 50 mV; then, the peaks started to be deformed. Thus, the value of 50 mV was chosen as optimum for all subsequent measurements.

#### Frequency

Changes of the value of frequency in a range of 20–100 Hz were investigated as the last key parameter of the SWV ramp. Figure 3c illustrates that height of APAP oxidation peak increases remarkably with higher frequencies, reaching its maximum at 40 Hz, setting frequency at higher values did not lead to anymore increase in peak current response. Hence, it was chosen as optimum value for entire analysis.

### Analytical performance of the method

**Fig. 4:**
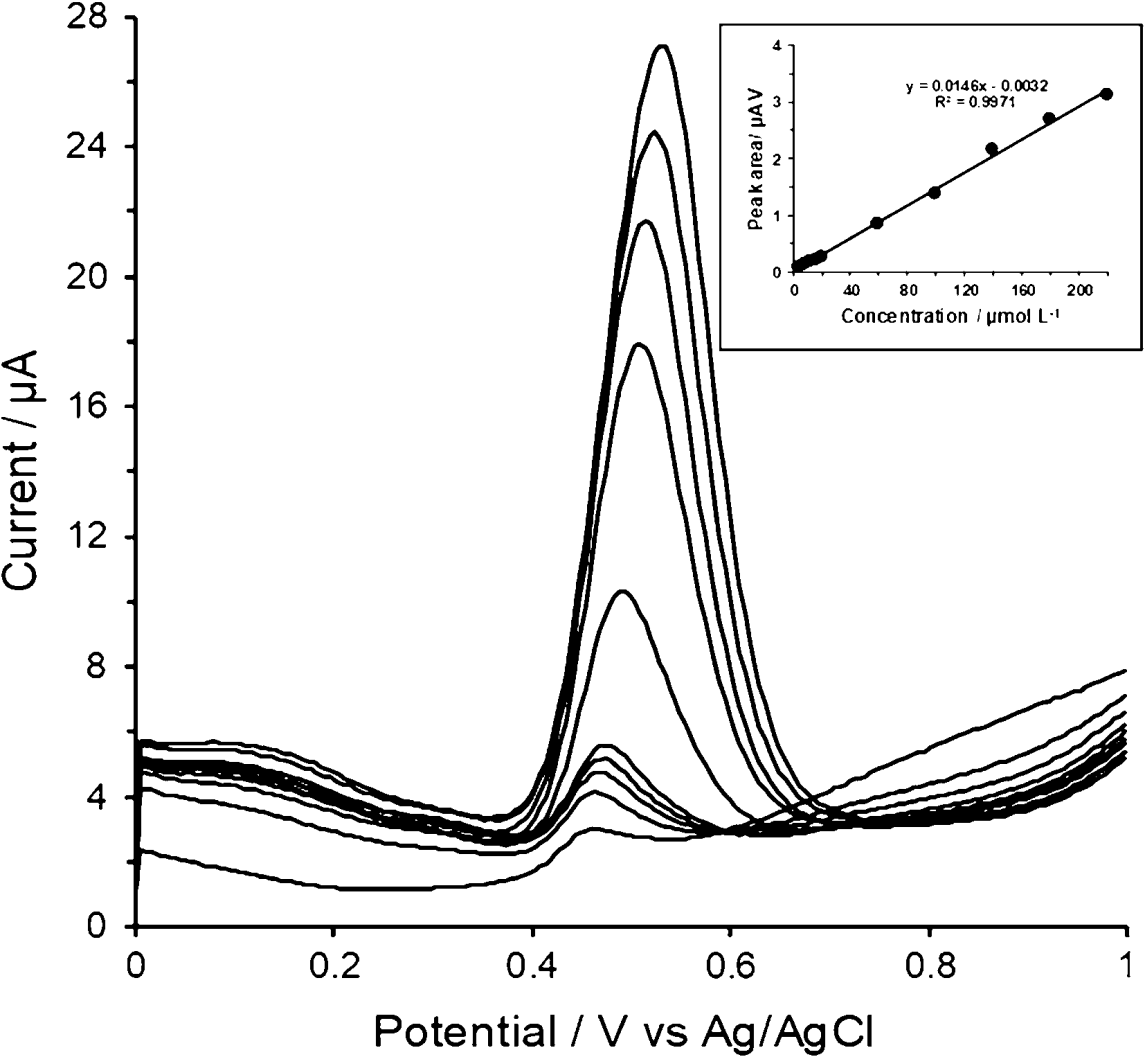
Voltammograms for 4, 8, 12, 16, 20, 60, 100, 140, 180 and 220 μmol L^−1^ APAP with corresponding calibration curve obtained at GCPE/RGO measured in 0.1 mol L^−1^ acetate buffer solution pH 5.0 using SWV at potential step 5 mV, potential amplitude 50 mV and frequency 40 Hz

The above-optimised experimental conditions and instrumental parameters had resulted in a set of conditions defining the new method whose analytical performance had to be examined experimentally. The calibration measurements were performed in a range of 1 × 10^–6^ to 5 × 10^–4^ mol L^−1^, resulting in a linearity of 4.0 × 10^–6^ to 2.2 × 10^–4^ mol L^−1^, defined by the regression equation *A*_p_(μA V) = 0.0146*c*(μmol L^−1^) − 0.0032, *R*^2^ = 0.9971 (see Fig. 4), limits of detection (LOD) and quantification (LOQ) to be 0.3 × 10^–6^ mol L^−1^ and 0.9 × 10^–6^ mol L^−1^, respectively, estimated according to the criteria LOD = 3* s*/*k* and for LOQ = 10* s*/*k* (where *k* refers to the slope of linear part of calibration curve specified above, *s* is the standard deviation of minimally five repetitions of a chosen concentration of 4 μmol L^−1^ APAP and with the average response of *I*_p_ = 0.0585 ± 0.0014 μA presented as arithmetic mean and the standard deviation). When testing the new method precision with respect to the repeatability of the signal of interest, a value ± 2.7% as the RSD was obtained. Before analyses of selected tablet-dosage forms, a series of recovery studies with a model sample were performed (with *n* = 5), yielding the satisfactory result of 103%.

### Interference study

Possible interferences were investigated for substances considered to represent typical minor constituents in the samples of interest—i.e., tablet formulation excipients (lactose monohydrate, magnesium stearate, croscarmellose sodium, corn starch) and main matrix of urine (uric acid, ascorbic acid, and urea). As found out, all these compounds did not exhibit any evident electrochemical signals within the working potential range applied, documenting a very good selectivity of the method developed. Finally, to complete the basic characterization of the new method, its comparison with other electrochemical approaches for the determination of APAP is surveyed in Table 1.

**Table 1:** An overview of reported electrochemical methods for paracetamol determination in biological fluid real samples and pharmaceutical formulations

Working electrodes	LOD (μmol L^−1^)	Linear range (µmol L^−1^)	*R*^2^	References
SPCE/NFG/tyrosinase–GTA/Nafion	0.5	1.4–70 and 3.8–130	0.998 and 0.997	[5]
CPE	–	1–100	0.996	[11]
PANI–MWCNTs	0.25	1–100 and 250–2000	–	[12]
Pd/Al	5	100–3000	–	[13]
PEDOT/GCE	1.13	2.5–150	0.996	[14]
SPE/PEDOT	1.39	4–400	0.993	[15]
CA/SPCEs	13	up to 2000	–	[16]
AQMCPE	0.13	5–150	0.998	[17]
GCPE/RGO	0.307	4–220	0.997	This work

*SPCE* screen-printed carbon electrode, *NFG* non-functionalized graphene, *GTA* glutaraldehyde, *CPE* carbon paste electrode, *PANI–MWCNTs* polyaniline–multi-walled carbon nanotubes, *Pd/Al* aluminum electrode modified by thin layer of palladium, *PEDOT* poly(3,4-Ethylenedioxythiophene, *GCE* glassy carbon electrode, *SPE* screen-printed electrode, *CA* cellulose acetate, *AQMCPE* anthraquinone modified carbon paste electrode

### Testing the reference RP–HPLC method before its use for validation

**Fig. 5:**
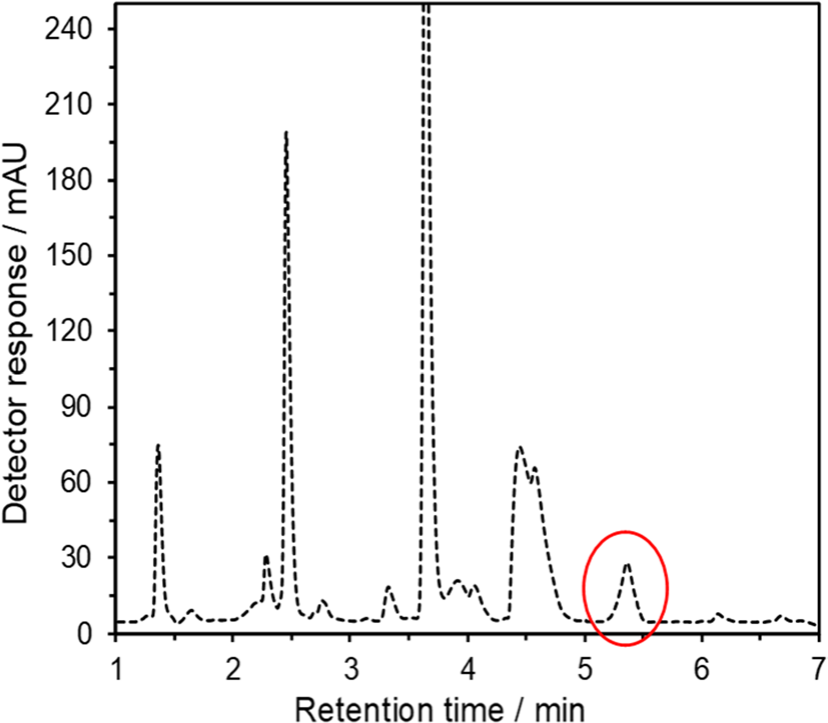
HPLC analyses of sample APAP in human urine. Ascentis Express C18 column (150 × 3 mm, 2.7 μm); mobile phase, 0.3% formic acid in water (**a**) and methanol (**b**); gradient program from 10 to 35% B for 10 min; flow rate, 0.5 mL min^−1^; sample volume, 5 μL; temperature, 30 °C; detection at 243 nm

The reference chromatographic procedure had to be yet validated before analyzing the samples intended to be suitable for the new voltammetric method—i.e., tablet dosage forms and urine samples. When using chromatographic conditions under Experimental in the respective section, it was found that the peak of APAP could be obtained at retention time of 5 min. 15 s. A typical chromatogram in Fig. 5 shows the respective calibration curve defined by the equation A(mAU) = 2,092.78*c*(μmol L^−1^) − 12,036.26 (*R*^2^ = 0.9972). Precision of the reference method was determined using five repeated measurements (*n* = 5), resulting in RSD of 0.79% for a urine sample collected from 5 healthy volunteers. Table 2 shows that the proposed method provides statistically satisfied results comparable to the chosen reference HPLC method as well as with the declared amount by a manufacturer.

**Table 2:** Comparison of SWV with standard HPLC method and declared APAP concentration in analysis of model sample, tablet dosage forms, and urine real sample

Sample	SWV	HPLC	Declared
Model	82.11 ± 2.76 μmol L^−1^	79.38 ± 0.79 μmol L^−1^	80 μmol L^−1^
Panadol® 500 mg	496.20 ± 5.59 mg per pill	495.63 ± 0.99 mg per pill	500 mg per pill
Human urine	15.05 ± 2.87 mg per 100 ml	14.67 ± 0.40 mg per 100 ml	–

Values given as confidence intervals *x̄* ± *st*_1−*α*_, where *x̄* is the arithmetic mean, *s* the standard deviation, and *t*_1−*α*_ the critical values (2.015 and 2.353) of Student’s *t* distribution for 5 and 3 repetitions of each analysis at *α* = 0.05, respectively

## Conclusions

Based on the above presentation of all the important features and characteristics of the newly developed method, one can state that it can offer a simple and quick approach of how to accurately and precisely determine acetaminophen (APAP) in pharmaceutical preparations and in urine samples. The developed procedure is based on the direct voltammetric oxidation of this substance at the RGO/GCPE in 0.1 mol L^−1^ acetate buffer (pH 5.0) as the supporting electrolyte of choice.

It seems that the method, for the first time described herein, could be offered as an interesting contribution for laboratories equipped with electrochemical instrumentation, or even for those equipped with chromatographic techniques that still represent the prevailing tool for determination of APAP in pharmaceutical and biological samples. In such cases, this new voltammetric procedure could serve as an independent and fully reliable reference method to occasionally check the results being obtained by routine chromatographic analyses.

## Supplementary Information

Below is the link to the electronic supplementary material.

Supplementary file1 (TIF 96 KB)

Supplementary file2 (TIF 107 KB)

Supplementary file3 (TIF 71 KB)
