# Supplementary figures and images for: Voltammetric determination of acetaminophen in pharmaceutical preparations and human urine using glassy carbon paste electrode modified with reduced graphene oxide
Source: Anal Sci. 2022 Jul 9;38(9):1213–20. doi: 10.1007/s44211-022-00150-2 (PMC9420686; doi:10.1007/s44211-022-00150-2)

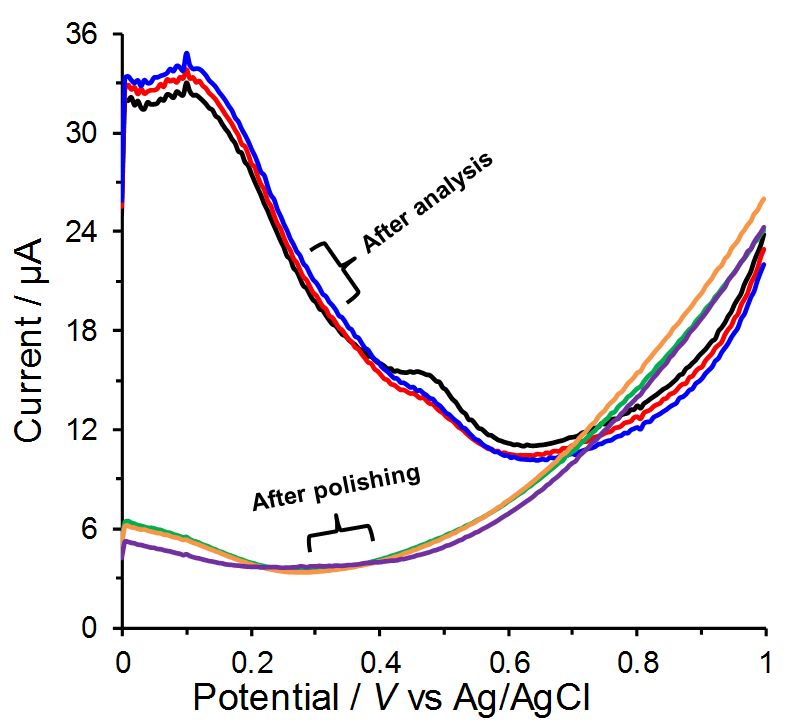

Supplement: Supplementary file 1 — Supplementary file1 (TIF 96 KB) [file 44211_2022_150_MOESM1_ESM.tif]

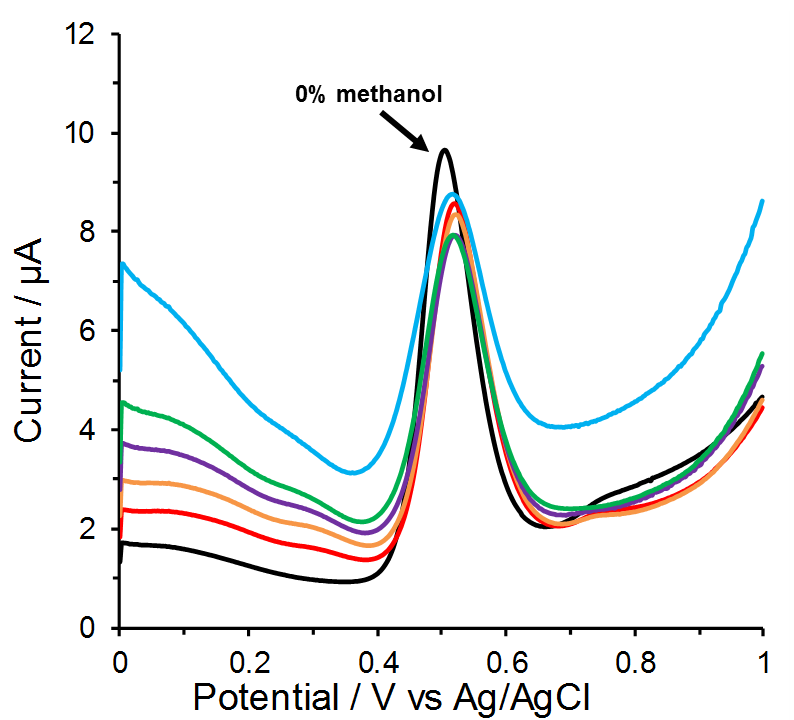

Supplement: Supplementary file 2 — Supplementary file2 (TIF 107 KB) [file 44211_2022_150_MOESM2_ESM.tif]

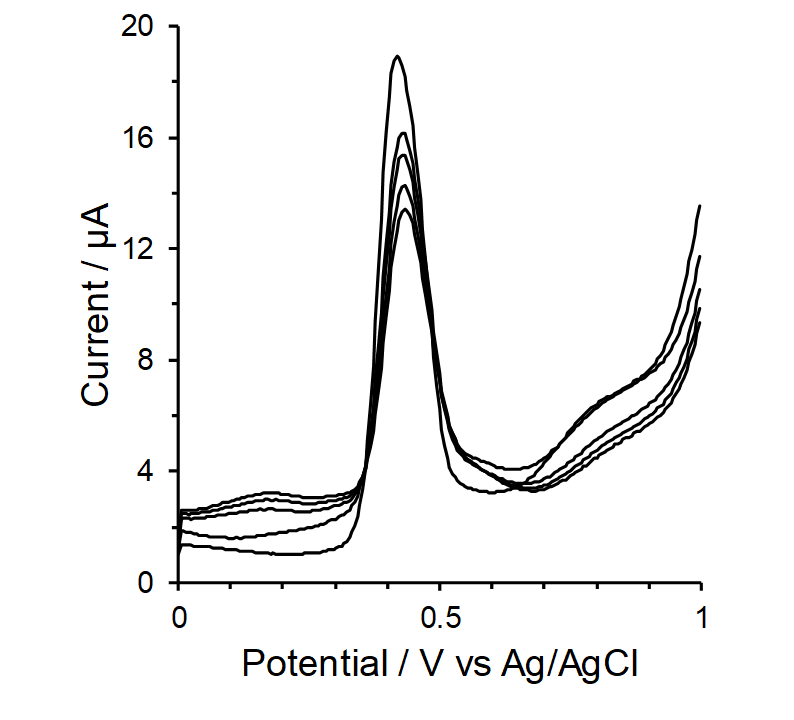

Supplement: Supplementary file 3 — Supplementary file3 (TIF 71 KB) [file 44211_2022_150_MOESM3_ESM.tif]
